# RhoA vesicle trafficking–mediated transglutaminase 2 membrane translocation promotes IgA1 mesangial deposition in IgA nephropathy

**DOI:** 10.1172/jci.insight.160374

**Published:** 2023-10-09

**Authors:** Zhong Zhong, Zhijian Li, Yanjie Li, Lanping Jiang, Qingyu Kong, Wei Chen, Shaozhen Feng

**Affiliations:** 1 Department of Nephrology, The First Affiliated Hospital, Sun Yat-sen University, Guangzhou, China.; 2 NHC Key Laboratory of Clinical Nephrology (Sun Yat-Sen University) and Guangdong Provincial Key Laboratory of Nephrology, Guangzhou, China.; 3 Zhongshan School of Medicine, Sun Yat-sen University, Guangzhou, China.

**Keywords:** Nephrology, Chronic kidney disease, Molecular biology, Molecular pathology

## Abstract

Transglutaminase 2 (TGase2) has been shown to contribute to the mesangial IgA1 deposition in a humanized mouse model of IgA nephropathy (IgAN), but the mechanism is not fully understood. In this study, we found that inhibition of TGase2 activity could dramatically decrease the amount of polymeric IgA1 (pIgA1) isolated from patients with IgAN that interacts with human mesangial cells (HMC). TGase2 was expressed both in the cytosol and on the membrane of HMC. Upon treatment with pIgA1, there were more TGase2 recruited to the membrane. Using a cell model of mesangial deposition of pIgA1, we identified 253 potential TGase2-associated proteins in the cytosolic fraction and observed a higher concentration of cellular vesicles and increased expression of Ras homolog family member A (RhoA) in HMC after pIgA1 stimulation. Both the amount of pIgA1 deposited on HMC and membrane TGase2 level were decreased by inhibition of the vesicle trafficking pathway. Mechanistically, TGase2 was found to be coprecipitated with RhoA in the cellular vesicles. Membrane TGase2 expression was greatly increased by overexpression of RhoA, while it was reduced by knockdown of RhoA. Our in vitro approach demonstrated that TGase2 was transported from the cytosol to the membrane through a RhoA-mediated vesicle-trafficking pathway that can facilitate pIgA1 interaction with mesangium in IgAN.

## Graphical abstract

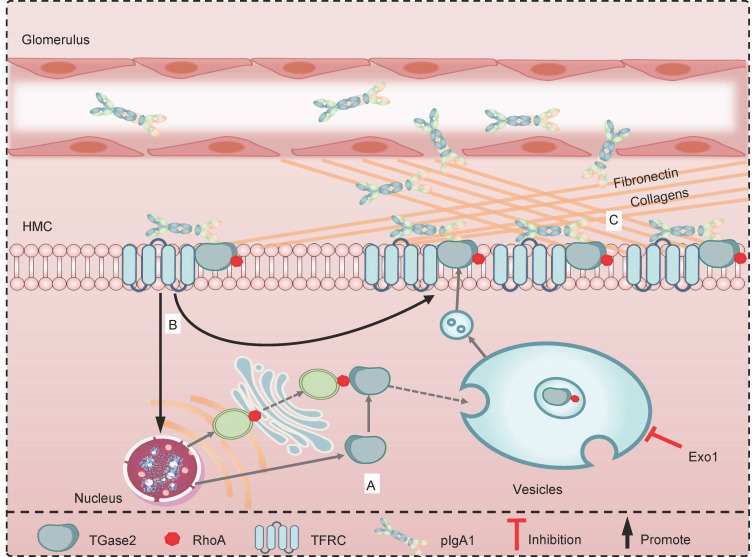

## Introduction

IgA nephropathy (IgAN) is the most common primary glomerulonephritis and a principal cause of kidney failure in China (1). It is characterized by predominant IgA deposition in the mesangial area of glomeruli. The mesangial IgA is exclusively of the IgA1 subclass and is polymeric and deficient in galactose (2, 3). The deposition of aberrantly glycosylated polymeric IgA1 induced various histopathological lesions, including mesangial cell proliferation, cytokines secretion, and extracellular matrix (ECM) production (4–6). Although the cause of the polymeric IgA1 deposition is still under debate, it has been confirmed that circulating IgA1 or its related complexes play an important role in the pathogenesis of IgAN (7, 8). Circulating soluble CD89 (sCD89) was found be to an essential part of IgA1 complexes and their deposition on mesangium, since IgA1 could not be deposited in the glomeruli without expressing human sCD89 in a mouse model (9, 10). In the humanized mouse model of IgAN (α1-KI/CD89-Tg), Berthelot et al. found that strong overexpression of mesangial transglutaminase 2 (TGase2) was colocalized with IgA1 deposition and that the absence of TGase2 could dramatically impair mesangial IgA1-sCD89 deposits and abolish hematuria (10), indicating the substantial role of TGase2 in IgA1 deposition. TGase2 may be responsible for a pathogenic amplification loop that facilitates IgA1-sCD89 deposition and mesangial cell activation in IgAN (11). However, there is no direct evidence to support this hypothesis.

TGase2 is a calcium-dependent enzyme and is involved in cross-linking proteins through the formation of ε-(γ-glutamyl) lysine bonds, which are stable and highly resistant to proteolytic degradation and mechanical challenge (12, 13). TGase2 acts as a structural protein and plays multifunctional roles in several cellular activities, such as cell-to-ECM interaction, cell growth, differentiation, and apoptosis (14, 15). TGase2 is found in different cell compartments, including the nucleus, cytoplasm, mitochondria, cell membrane, and the ECM (12, 16–19), and its subcellular localization is an important determinant of its function. Despite the fact that a link between TGase2 and the IgA1 complex or IgA1 was well established in the mouse model or in vitro protein-to-protein interaction (PPI) without cell culture (10), the expression pattern of TGase2 in human mesangial cells, and the pathway by which TGase2 is transferred from cytosol to the membrane during mesangial IgA1 deposition, have not been fully elucidated.

Since TGase2 lacks the leader signal peptide (20, 21) that is essential for protein secretion from living cells by the classical endoplasmic reticulum/Golgi pathway, it is of great interest to investigate the role of TGase2 in mesangial IgA1 deposition and the signal pathway regulating TGase2 during IgA1 deposition in glomerular mesangium. Therefore, this study aimed to discover the mechanism underlying TGase2-mediated regulation of mesangial IgA1 deposition in IgAN.

## Results

### Reduction of polymeric IgA1 binding to HMC by inhibiting TGase2 activity.

In order to explore the effect of TGase2 on the mesangial deposition of IgA1, serum IgA1 was firstly isolated from patients with IgAN by Jacalin affinity chromatography (Supplemental Figure 1, A and B; supplemental material available online with this article; https://doi.org/10.1172/jci.insight.160374DS1). Polymeric IgA1 (pIgA1) was then separated from monomeric IgA1 (mIgA1) by size chromatography (Supplemental Figure 1, C and D) and confirmed by native PAGE and Western blot (Supplemental Figure 1, E and F). Purified pIgA1 from patients with IgAN exhibited significantly high binding capacity to the human mesangial cell line (HMC), while mIgA1 from patients with IgAN, as well as mIgA1 and pIgA1 from normal controls, showed low or no binding capacity to HMC (Supplemental Figure 2A). Furthermore, in vitro cell culture results indicate that pIgA1 from patients with IgAN could significantly dose-dependently increase the proliferation of HMC (Supplemental Figure 2B) as well as induce the expression levels of fibronectin (Fn), collagen type I (Col-I) (Supplemental Figure 2C). Thus, purified pIgA1 from patients with IgAN was used to further investigate the effect and mechanism of TGase2 on mesangial IgA1 interaction.

**Figure 1:**
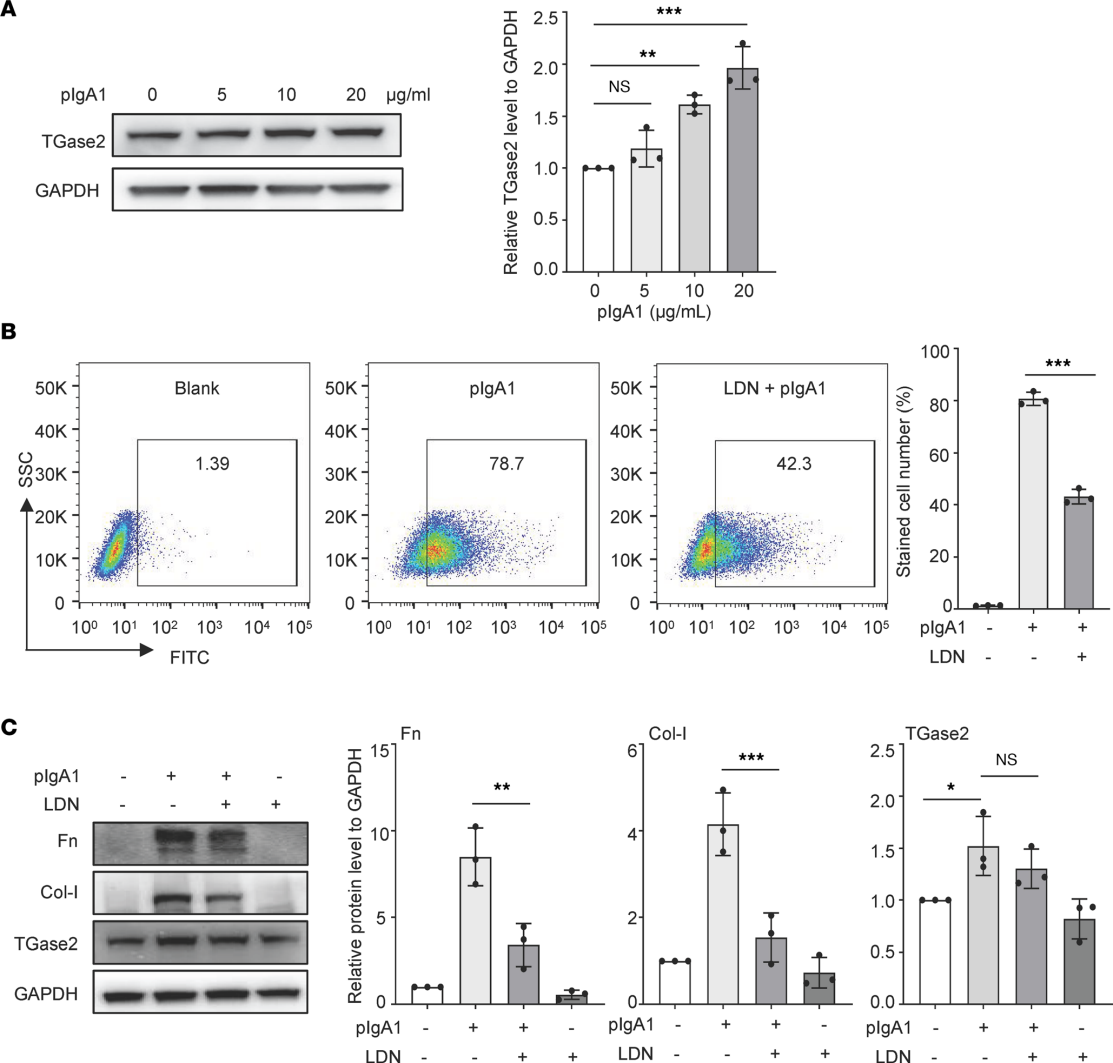
Effect of TGase2 inhibition on reducing pIgA1 binding to HMC. (**A**) Western blot analysis of TGase2 expression in HMC after various concentration of pIgA1 stimulation for 48 hours. Quantification is shown in the bar graph as mean ± SD (*n* = 3, 1-way ANOVA). (**B**) HMC (1.6 × 10^5^ cells in 200 μL 0.5% FBS-1640 medium) was preincubated with or without 10 μM LDN-21279 (LDN) at 37°C for 1 hour and then treated with pIgA1 from patients with IgAN (10 μg/mL) for another 1 hour. The stained cells were analyzed on FACScan. Representative plots and bar graph show the percentages of IgA1 staining cells. Data represent the mean ± SD (*n* = 3). Significant difference was determined by 1-way ANOVA. (**C**) Western blot analysis of fibronectin (Fn), collagen type I (Col-I), and TGase2 expression in HMC, which were pretreated with LDN-27219 (LDN, 10 μM) for 1 hour, followed by pIgA1 (10 μg/mL) treatment for 48 hours. Data are shown as mean ± SD (*n* = 3, 1-way ANOVA). *P < 0.05, **P < 0.01, ***P < 0.001.

The expression of TGase2 was dose-dependently upregulated in HMC with pIgA1 stimulation (Figure 1A). In order to investigate the influence of TGase2 on pIgA1 deposition on HMC, the activity of TGase2 was inhibited using LDN-27219, a specific TGase2 inhibitor. Flow cytometry analysis revealed that the cell survival of HMC was not significantly affected by addition of 10 μM or less of LDN-21279 (Supplemental Figure 3). However, preincubation of LDN-21279 (10 μM) significantly decreased the amount of pIgA1 binding to HMC when compared with the addition of pIgA1 alone (Figure 1B). Immunostaining results also confirmed that inhibition of TGase2 by LDN-21279 could dramatically reduce the amount of pIgA1 deposition to HMC (Supplemental Figure 4). Moreover, the expression levels of Fn and Col-I, which were induced by pIgA1, were significantly decreased by preincubation of HMC with LDN-21279 (Figure 1C). Although TGase2 is known to be tightly involved in the stabilization of ECM (13), it is postulated that the TGase2 inhibitor might decrease the ECM synthesis via reducing the amount of pIgA1 interacting with HMC. TGase2 may be essential for pIgA1 depositing on mesangial cells.

### TGase2 expression in the cytosol and membrane of HMC.

**Figure 2:**
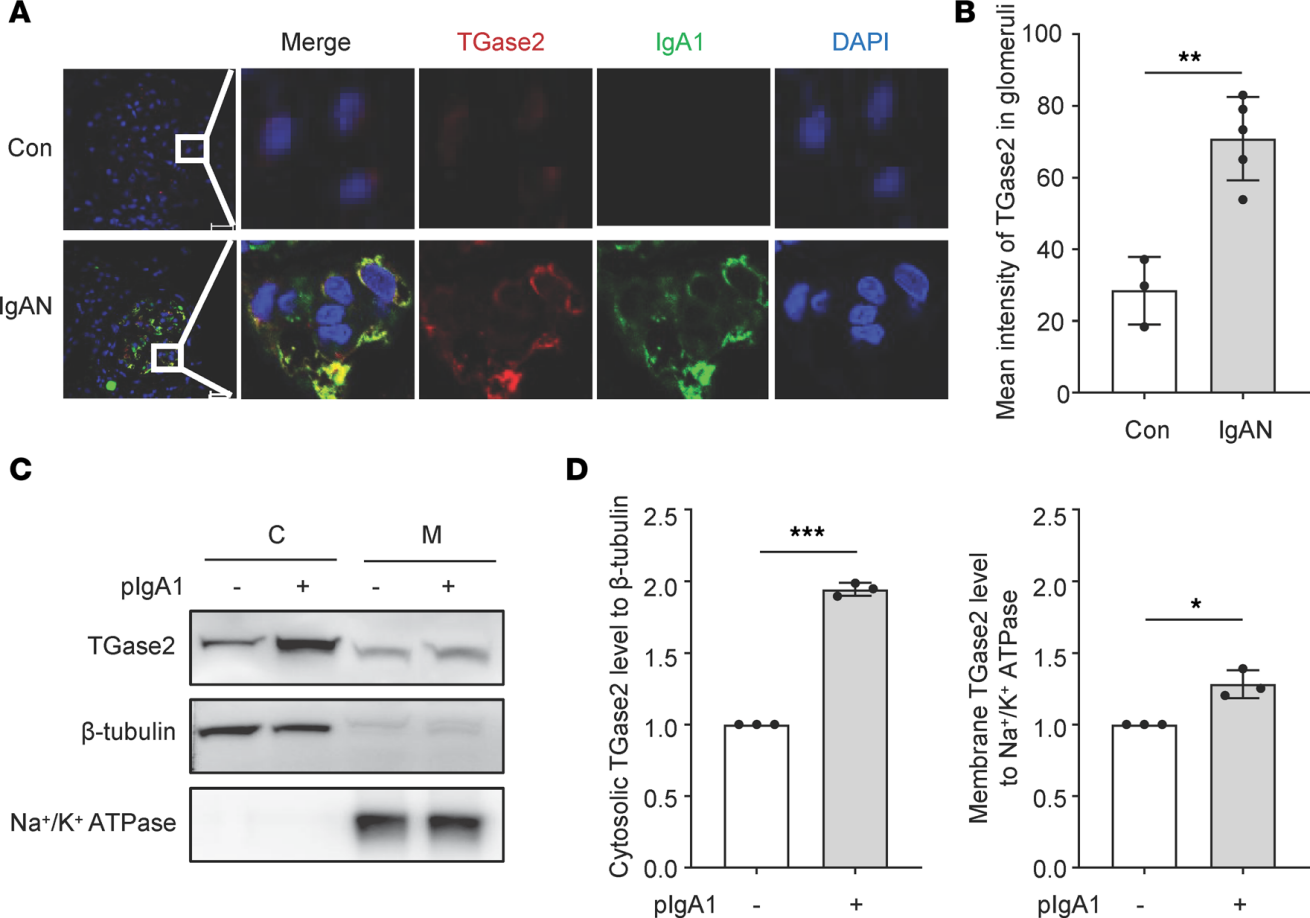
Identification of TGase2 expression in the membrane of human mesangial cells. (**A**) Representative immunofluorescence staining of TGase2 (red) and IgA1 (green) in the kidney of normal controls (Con, *n* = 3) and patients with IgAN (IgAN, *n* = 5). Total original magnification, ***×***630. (**B**) Fluorescence intensity was analyzed by ImageJ software. Bars represent the mean fluorescence intensity of TGase2 signals in glomeruli from at least 5 visual fields (***×***40) of each sample. Data are shown as mean **±** SD. Statistical analysis between groups was performed by unpaired 2-tailed Student *t* test. (**C**) Western blot analysis of TGase2 expression in HMC with or without pIgA1 (10 μg/mL) treatment for 48 hours. Cytosolic (C) and membrane (M) fractions were validated for enrichment of cytosol marker (β-tubulin) or membrane marker (Na^+^/K^+^ ATPase). (**D**) Quantification of **C** is shown in the bar graph; data are shown as mean ± SD (*n* = 3, 1-way ANOVA). *P < 0.05, **P < 0.01, ***P < 0.001.

Immunostaining results show that specific signals of TGase2 were upregulated and colocalized with IgA1 in the kidney mesangium of patients with IgAN compared with that in normal controls (Figure 2, A and B, and Supplemental Figure 5). Since TGase2 inhibition could reduce the amount of pIgA1 interacting with HMC (Figure 1), the expression pattern of TGase2 was further determined. An in vitro cell model revealed that TGase2 was expressed either in the cytosol or on the membrane of HMC, and this was validated by detection of cytosol marker β-tubulin and membrane marker Na^+^/K^+^ ATPase (Figure 2C). There was an increase in cytosolic and membrane expression of TGase2 after pIgA1 treatment (Figure 2, C and D).

### TGase2 interaction networks reveal vesicular trafficking and actin dynamics proteins involved in pIgA1 deposition.

**Figure 3:**
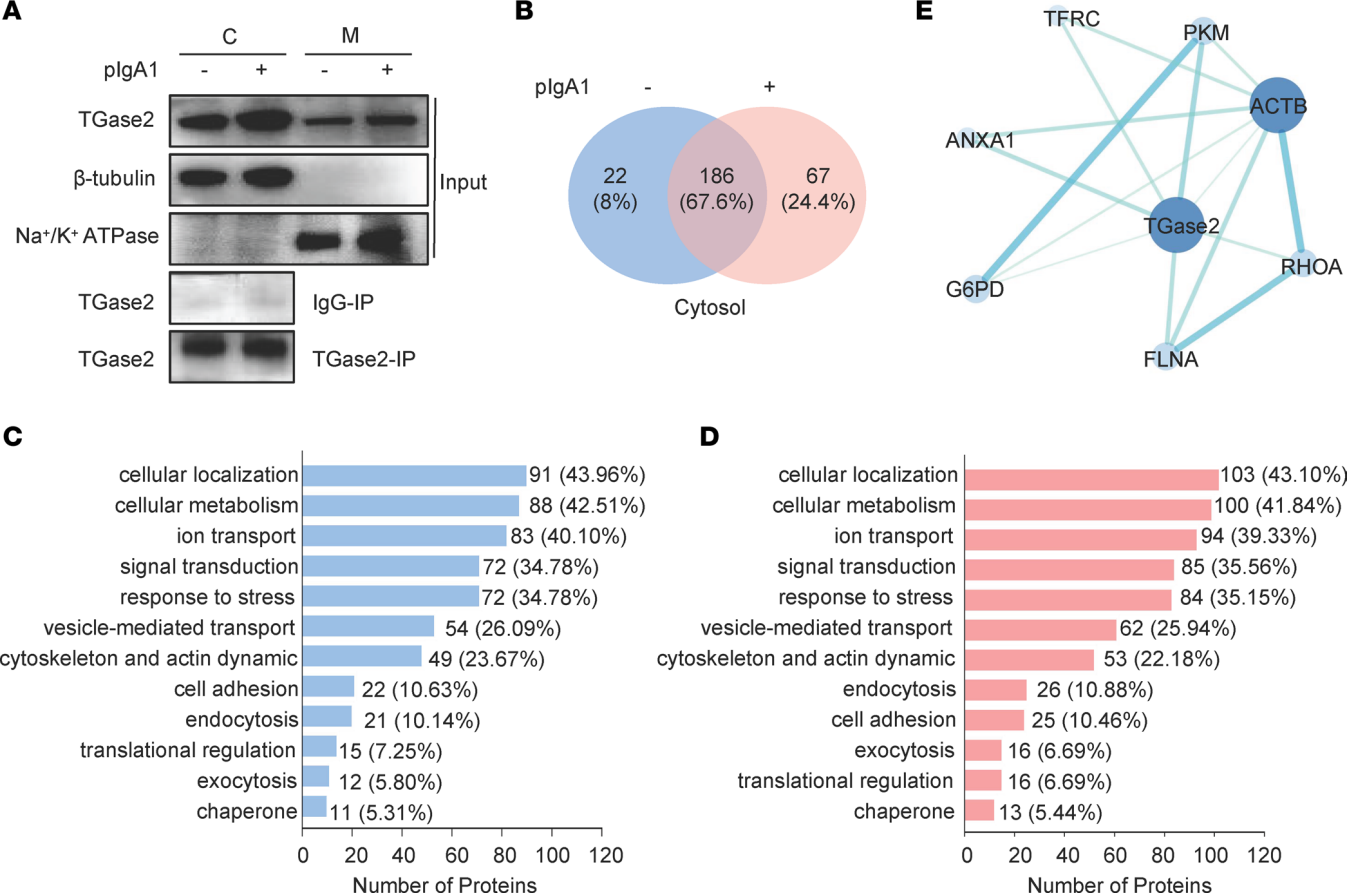
TGase2 interactome in the cytosol of human mesangial cells treated with pIgA1. (**A**) HMC were treated with or without pIgA1 (10 μg/mL) for 48 hours. Cytosolic (C) and membrane (M) fractions were separated and validated for enrichment of β-tubulin and Na^+^/K^+^ ATPase by Western blot. TGase2-associated proteins in the cytosol were immunoprecipitated using anti-TGase2 antibody (*n* = 2). The isotype control antibody cross-link to protein A/G magnetic beads was used as a control (*n* = 2). (**B**) The TGase2-associated proteins after IP were analyzed via MS. The number of proteins was identified as specifically associated with TGase2 after exclusion of nuclear, mitochondrial, and ribosomal proteins. (**C** and **D**) Functional distribution of TGase2-associated proteins from cytosolic fractions without (**C**) or with pIgA1 deposition (**D**). Proteins were clustered based on their functions in Homo sapiens, which were determined by protein identification search in the UniProtKB database (https://www.uniprot.org/). Column charts display the distribution of different functions of TGase2-associated proteins with numbers and the percentage of proteins falling in the various functional categories. (**E**) The protein-interaction network was built from TGase2-associated proteins from cytosolic fraction without pIgA1 treatment (208 proteins) or with pIgA1 treatment (253 proteins). The protein-interaction network was mapped against the Homo sapiens reference database using the STRING tool. Candidates were selected using both known and predicted protein interactions with a threshold confidence level of 0.5. Networks were imported into Cytoscape software, and the direct partners of TGase2 were shown. The size and color shade of circles are proportional to the number of protein interactions, while the thickness and color shade of the lines are proportional to the confidence of the interactions. Here, only 1 interactome of cytosolic TGase2 is shown because it remained the same in HMC without or with pIgA1 treatment.

We predicted the presence of signal peptide by SignalP 6.0 (https://services.healthtech.dtu.dk/services/SignalP-6.0/), and the result revealed no signal peptide at the N-terminus of the precursor protein sequence of human TGase2 (GenBank: AAB95430.1, https://www.ncbi.nlm.nih.gov/protein/AAB95430.1). We proposed that cytosolic TGase2 probably transfers to the membrane in a nonclassical pathway. The protein IP assay with anti-TGase2 antibody combined with mass spectrometry (MS) were used to identify the putative interacting proteins associated with cytosolic TGase2. The results of Western blotting confirmed the presence of TGase2 in the cytosol fractions of HMC (Figure 3A). Identification of the interacting proteins by MS revealed that most of cytosol TGase2-associated proteins (67.6%, 186 of 275) were overlapped in HMC with and without pIgA1 deposition (Figure 3B). Both TFRC and TGase2 could be found in the overlapping proteins. The complete lists of cytosol proteins associated with TGase2 in HMC with or without pIgA1 deposition are shown in Supplemental Tables 1 and 2. Function analysis obtained from UniprotKB database (https://www.uniprot.org) revealed that, in HMC with or without pIgA1 treatment, enrichment of TGase2-associated proteins involved in cellular localization, metabolism, ion transport, signal transduction, vesicle-mediated transport, cytoskeleton and actin dynamic, and other physiological activities (Figure 3, C and D). There was a slight increase in the proportion of proteins associated with TGase2 involved in exocytosis after pIgA1 stimulation (5.8% versus 6.69%).

Then, the protein interaction network was built from TGase2-associated proteins from cytosolic fraction of HMC without pIgA1 treatment (208 proteins; Supplemental Table 1) and with pIgA1 treatment (253 proteins; Supplemental Table 2), respectively. The network of TGase2 interactomes was obtained from the STRING database v11, which was based on both known and predicted PPI and further modified using Cytoscape software. Both in cytosol of HMC with and without pIgA1 treatment, TGase2 was predicted to directly interact with Transferrin receptor protein 1 (TFRC), Annexin A1 (ANXA1), Filamin-A (FLNA), Ras homolog family member A (RhoA), Actin cytoplasmic 1 (ACTB), Pyruvate kinase M (PKM), and Glucose-6- phosphate 1-dehydrogenase (G6PD) (Figure 3E and Supplemental Figures 6 and 7). IgA1 receptor TFRC was reported to interact with TGase2 in the literature (9, 22), indicating the reliability of this PPI-based prediction.

### TGase2 transfers to membrane and regulates pIgA1 deposition through a vesicular trafficking pathway.

**Figure 4:**
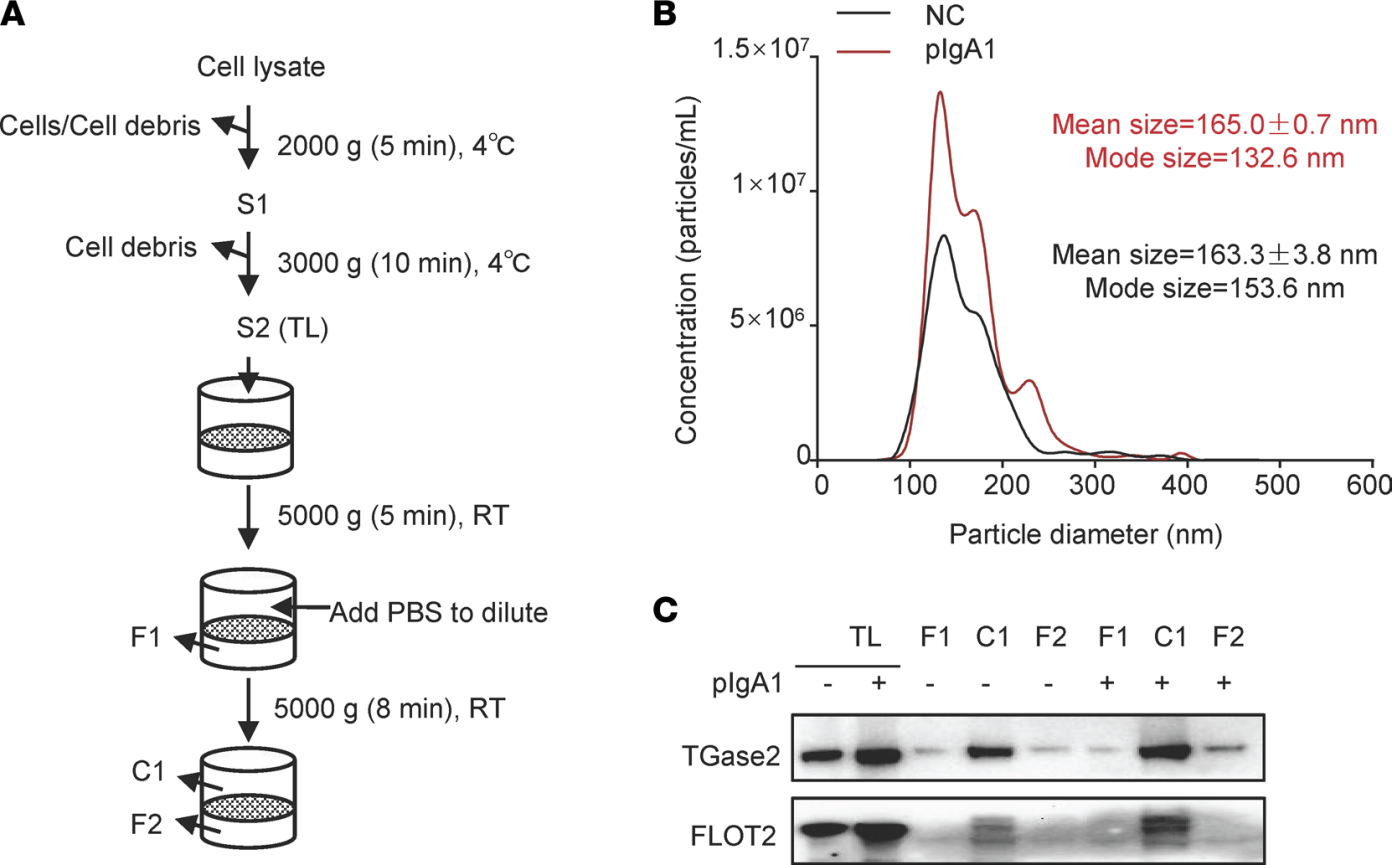
TGase2 is present in cellular vesicles of plasma membrane origin in HMC. (**A**) Flowchart of the centrifugation and filtration steps for the isolation of cellular vesicles. S1 and S2 indicate supernatants. S2 is total lysate (TL). C1 indicates purified cellular vesicles. F1 and F2 indicate filtrate. (**B**) HMC were planted in a 6-well plate overnight and then grown in serum-free medium with supplementation of 10 μg/mL pIgA1 or PBS for 24 hours. Cellular vesicles were purified as shown in **A**. The microparticle size distribution in fractions C1 from HMC treated with PBS (NC) or pIgA1 was obtained using nanoparticle tracking analysis (*n* = 3). (**C**) Expression of FLOT2 and TGase2 in purified cellular vesicles (C1), filtrate fractions (F1 and F2), and cell lysate (TL) from HMC treated with or without pIgA1 were measured by Western blot in equal amounts of proteins from different fractions.

Cellular vesicles were isolated from HMC using filtration (Figure 4A) and characterized by tunable resistive pulse sensing analysis with a NanoSight NS300 instrument. The amount of cellular vesicles was increased after pIgA1 treatment (Figure 4B). The mean size of cellular vesicles from HMC was about 163 nm. Densitometric analysis of TGase2 relative to vesicle marker flotillin-2 (FLOT2) revealed an enrichment of TGase2 in the cellular vesicle (C1, the purified cellular vesicles fraction ) and a few amount of TGase2 in plasma (F1 and F2, the filtrate fraction) (Figure 4C). Moreover, pIgA1 treatment caused a trend of increase in TGase2 in the vesicle fraction (Figure 4C).

**Figure 5:**
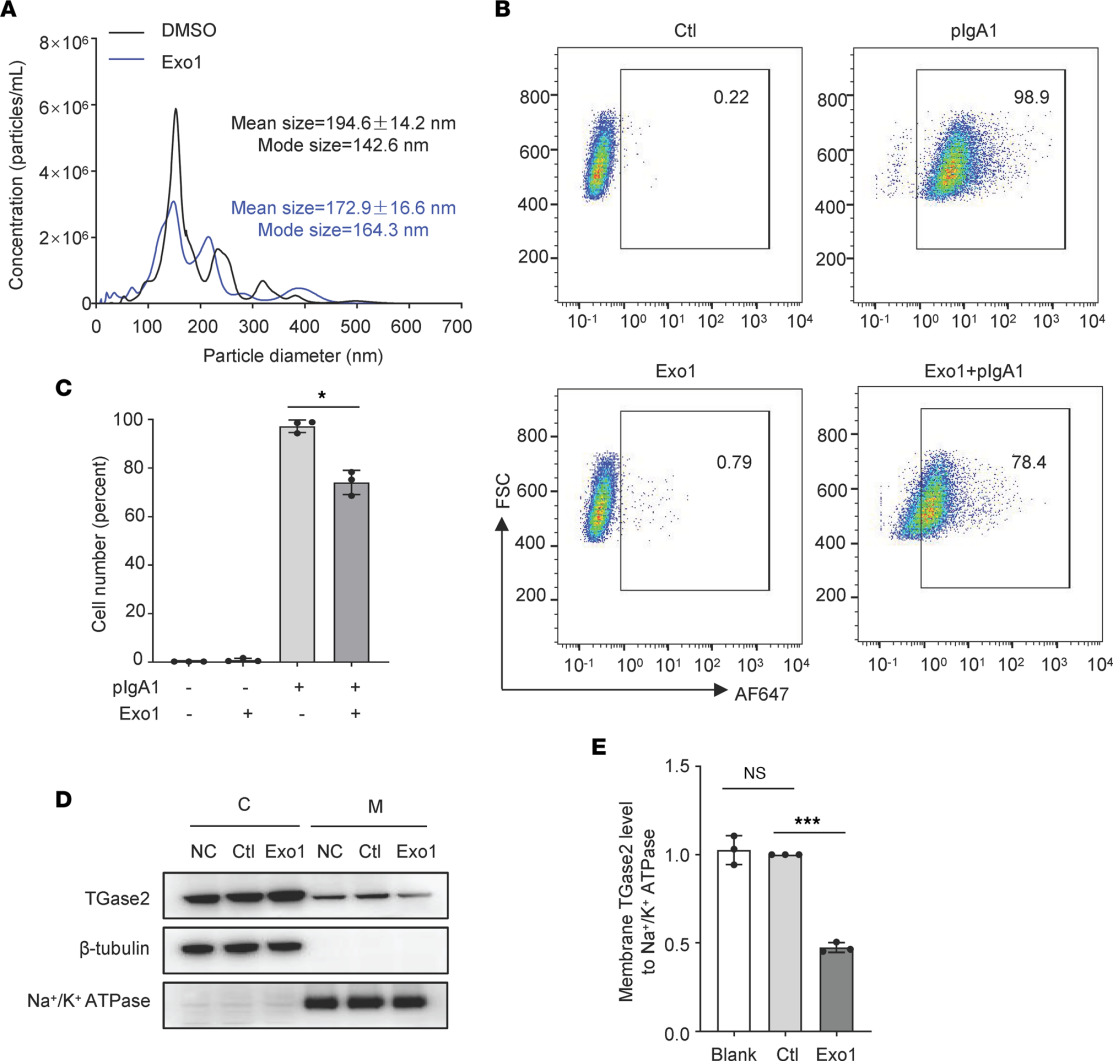
Polymeric IgA1 deposition on HMC and the membrane transfer of cytosol TGase2 are reduced by vesicular trafficking inhibition. (**A**) Exponentially growing HMC were cultured in serum-free medium with Exo1 (30 μM) or dissolve for 24 hours. Cellular vesicles were purified by centrifugation (5,000*g* for 5–8 minutes at room temperature) and filtration as shown in Figure 4. The microparticle size distribution of cellular vesicles was obtained using nanoparticle tracking analysis (*n* = 3). (**B**) HMC (1.6 × 10^5^ cells in 200 μL 0.5% FBS-1640 medium) was preincubated with 30 μM Exo1 or dissolve (Ctl) at 37°C overnight and then treated with pIgA1 (20 μg/mL) for 1 hour. Cells were washed with 0.5% BSA-PBS and then stained with goat anti–human IgA antibody. Cells were washed with 0.5% BSA-PBS and then stained with goat anti–human IgA antibody, followed by secondary staining with Alexa Fluor 647–conjugated donkey anti-goat antibody. Cells stained without anti-IgA antibody were used as negative control. The stained cells were analyzed on FACScan. (**C**) The results were expressed as mean ± SD of the percentage of stained cells from 3 individual experiments. Data were analyzed by 1-way ANOVA. (**D**) Exponentially growing HMC planted in a 6-well plate were treated with Exo1 (30 μM), dissolve (Ctl), or blank (NC) in serum-free medium. After 24 hours, Western blot was used to detect the expression of TGase2 in the cytosolic (C) and membrane (M) fraction. (**E**) Quantification of **D** is shown in the bar graph; data are shown as mean ± SD (*n* = 3, 1-way ANOVA). *P < 0.05, ***P < 0.001.

To determine the role of vesicular trafficking in TGase2-mediated pIgA1 interacting with HMC, we, therefore, investigated whether the inhibition of vesicular trafficking could affect pIgA1 deposition on HMC and the membrane transfer of cytosol TGase2. After treatment of HMC with vesicular trafficking inhibitor Exo1, the amount of cellular vesicles was reduced (Figure 5A). Both deposition of pIgA1 on HMC and the membrane expression of TGase2 were significantly decreased after Exo1 treatment (Figure 5, B–E).

### Vesicular trafficking of TGase2 depends on RhoA in HMC.

**Figure 6:**
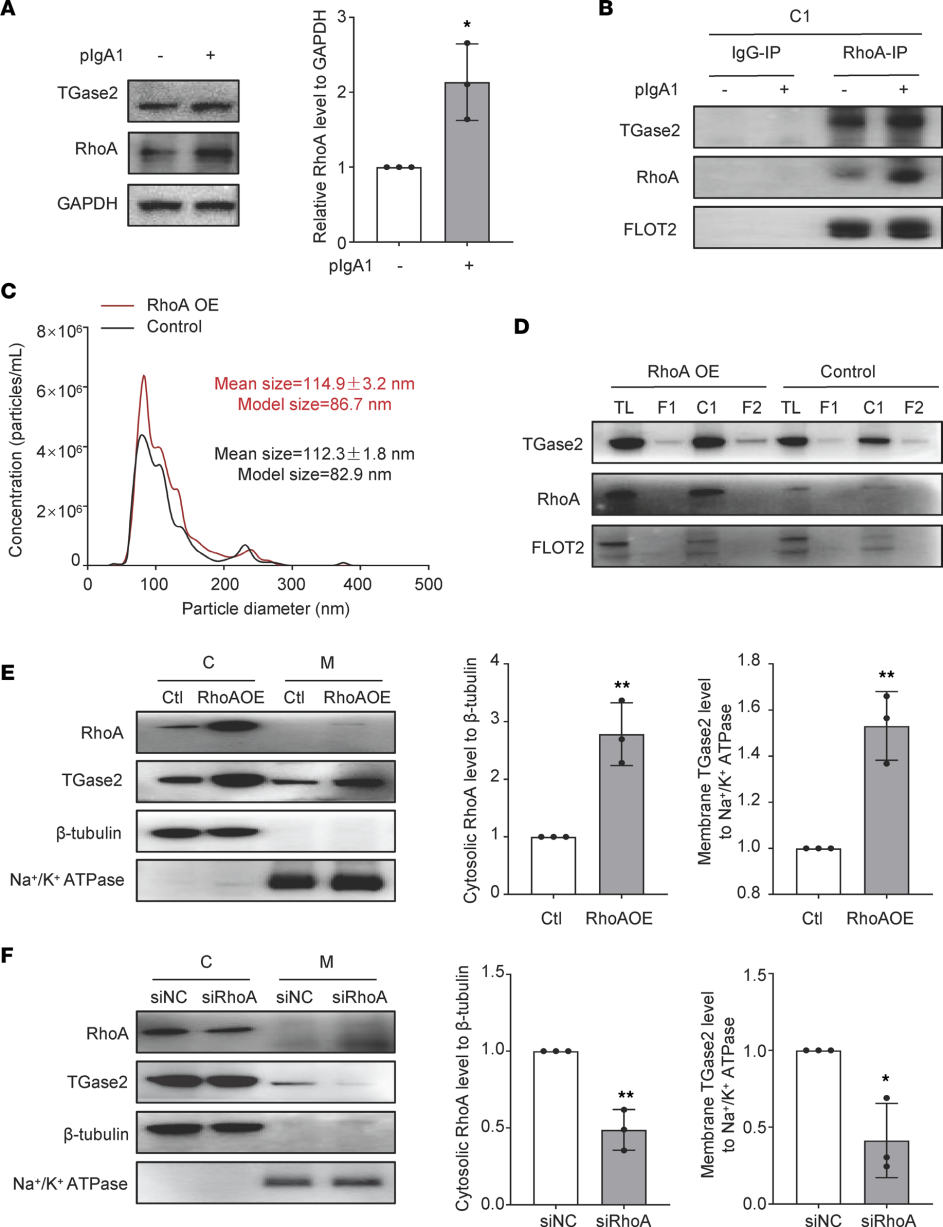
TGase2 transfers to membrane dependently on RhoA-mediated vesicle-trafficking in HMC. (**A**) Western blot analysis of RhoA in HMC with or without pIgA1 (10 μg/mL) treatment for 48 hours. Quantification is shown in the bar graph; data are shown as mean ± SD (n = 3, 2-tailed Student *t* test). (**B**) Cellular vesicles were purified from HMC with pIgA1 (10 μg/mL) or not for 48 hours by centrifugation and filtration as shown in Figure 4. RhoA was immunoprecipitated from vesicular fractions (C1) with rabbit anti-RhoA antibody. The presence of TGase2 and RhoA in immunoprecipitants were detected by Western blot (*n* = 2). (**C**) HMC were transiently transfected with RhoA overexpression plasmid (RhoAOE) or empty vector (Control) for 48 hours. Cellular vesicles were purified from as shown in Figure 4. The microparticle size distribution of cellular vesicles was obtained using nanoparticle tracking analysis (*n* = 3). (**D**) Lysates of vesicular fraction (C1) isolated from HMC with overexpression of RhoA obtained as described in **C**, total lysate (TL) and filtrate fractions (F1 and F2) were tested for RhoA, TGase2 and FLOT2 by Western blot in equal amounts of proteins from different fractions (*n* = 3). (**E**) Western blot analysis of RhoA and TGase2 in the cytosolic (C) and membrane (M) fractions of HMC which were transfected with RhoA overexpression plasmid (RhoAOE) or empty vector (control) for 48 hours. Quantification is shown in the bar graph as mean ± SD (*n* = 3, 1-way ANOVA). (**F**) Western blot analysis of RhoA and TGase2 in the cytosolic (C) and membrane (M) fractions in HMC which were transfected with siRNA targeting RhoA (siRhoA) or negative control (siNC) for 48 hours. Quantification is shown in the bar graph as mean ± SD (*n* = 3, 1-way ANOVA).*P < 0.05, ** *P* < 0.01.

Since RhoA was found to be present in the cytosol TGase2 interactome in HMC either with or without pIgA1 deposition (Figure 3E), and it has been reported that RhoA plays a role in regulating vesicular trafficking pathway (23, 24). The role of RhoA in regulating vesicular trafficking–associated membrane transport of cytosol TGase2 was further determined. Western blot results show that the RhoA expression level was significantly upregulated after pIgA1 stimulation (Figure 6A). IP of RhoA from vesicular lysates revealed that RhoA clearly pulled down TGase2 and there were more TGase2 immunoprecipitated by RhoA after pIgA1 treatment (Figure 6B), suggesting an interaction between TGase2 and RhoA within cellular vesicles, which was enhanced by the stimulation of pIgA1. Moreover, RhoA overexpression greatly increased the amount of cellular vesicles (Figure 6C) as well as TGase2 expression level in the cellular vesicle fraction (C1) (Figure 6D). Furthermore, the membrane levels of TGase2 were increased by overexpression of RhoA (Figure 6E), while they were reduced by knockdown of RhoA (Figure 6F). Taken together, cytosolic TGase2 was transported onto the membrane via RhoA-mediated vesicular trafficking pathway in mesangial cells, and pIgA1 treatment can induce more membrane transport of TGase2 through “RhoA positive” vesicles.

## Discussion

This is a qualitative analysis of TGase2-interacting proteins from an in vitro cell model of mesangial deposition of pIgA1 from patients with IgAN to identify the precise role and molecular mechanism of TGase2 in the interaction between pIgA1 and mesangial cells. TGase2-associated proteins were identified using an original targeted proteomic strategy by combining TGase2 IP from cytosol preparation of HMC with or without pIgA1 deposition.

The glomerular IgA eluted from tissue specimens of patients with IgAN is exclusively of the IgA1 subclass, predominantly in the polymeric form (3, 25). The observation of the frequent recurrence of IgAN in renal allografts suggests that the glomerular IgA1 is derived from the circulation (26–28). Therefore, we isolated polymeric IgA1 from primary patients with IgAN to investigate the role and regulatory mechanism of TGase2 in mesangial IgA1 deposition. As previously reported (6, 29, 30), we also found that purified pIgA1 from patients with IgAN interacted more efficiently with HMC than mIgA1 or pIgA1 from healthy controls and can induce the proliferation and ECM accumulation of cultured human mesangial cells. Until now, the pathogenetic importance of pIgA1 has been shown in several in vitro studies (31–33). The cultured human mesangial cells provide a convenient model for evaluating the biologic activities of pIgA1, as well as the molecular mechanism of mesangial IgA1 deposition.

Bioinformatics analysis of the cytosolic TGase2-associated proteins in this cell model revealed that proteins were mostly enriched in cellular localization, signal transduction, vesicle-mediated transport, and cytoskeleton and actin dynamics. The results of the PPI network show that direct interacting partners of TGase2, such as FLNA, ACTB, ANXA1, and RhoA, could link the actin filaments to membrane glycoproteins or regulate the actin cytoskeletal remodeling (23, 24, 34, 35). Rho proteins are well known for their effects on the actin cytoskeleton and are activated in response to a variety of extracellular stimuli (36). RhoA has been shown to regulate vesicle/membrane trafficking by controlling the actin cytoskeleton organization (23, 24). By exploiting IP in combination with MS, we have uncovered a pathway for the cellular export of TGase2 in mesangial cells. Cytosolic TGase2 is transferred to the membrane through a RhoA-mediated vesicle tracking pathway, and the membrane expression of TGase2 was upregulated due to the increased RhoA^+^ vesicles during pIgA1 depositing on HMC. Previous studies indicate that TGase2 can be transferred from cytosol to membrane and even secreted by different nonclassical secretory pathway mechanisms such as exosome (19, 37), endosome (38), and membrane pore formation (39). The occurrence of different nonclassical secretory pathway mechanisms could be related to the conditions triggering their release and the cell type (40).

In recent years, the multiple roles played by TGase2 have been widely elucidated, defining it as a multifaceted protein with opposite activities both in physiological and pathological conditions. The activities mediated by TGase2 can be influenced by its altered cellular localization. Although TGase2 is mainly expressed in the cytoplasm, it has also been detected in the extracellular space within exosomes (19) or in the association with proteins of ECM (41) or the cell membrane, as observed in HMC. As a result, TGase2 can influence several and even opposing processes, such as tissue stability, cell proliferation and differentiation, cell adhesion and migration, cell programmed death, and autophagy (42).

TGase2, a ubiquitous enzyme, is well known for its involvement in the development of renal fibrosis (19, 43). Furini et al. revealed that the secretion of TGase2 from tubular epithelial cells into the surrounding interstitium promoted fibrotic remodeling during renal fibrosis progression (19). Besides renal fibrosis, another research field of TGase2 in renal disease is glomerular pathology of IgAN. Ikee et al. showed that TGase2 was largely overexpressed in the glomeruli of patients with IgAN, and its expression was correlated with the severity of clinical and histological lesions (44). Our results also show that TGase2 immunostaining was upregulated in the glomeruli of patients with IgAN compared with controls with normal kidney function. What’s more, we confirmed that TGase2 was colocalized with IgA1 in patients with IgAN. In vitro cell culture of HMC, cytosolic, and membrane TGase2 expression was significantly increased with pIgA1 deposition. Interestingly, inhibition of TGase2 activity remarkably reduced the amount of pIgA1 interacting with HMC, resulting in less ECM protein production. Membrane TGase2 could interact with pIgA1 and TFRC in HMC. Our results further explain that mesangial IgA1-sCD89 deposits were dramatically impaired by KO TGase2 in a humanized mouse model for IgAN (10), probably due to the absence of connector TGase2 between IgA1-sCD89 and TFRC.

**Figure 7:**
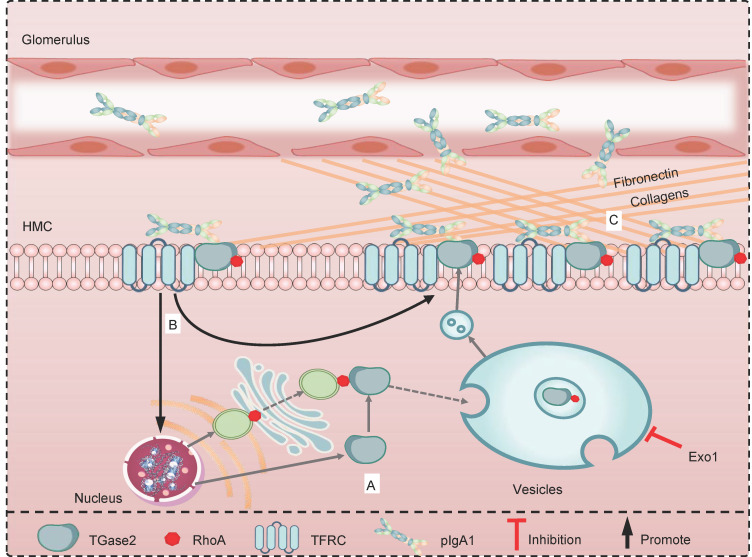
The proposed pathway of TGase2 from cytosol to membrane through RhoA-mediated vesicle trafficking promotes pIgA1 deposition on human mesangial cells. (**A**) TGase2 is weakly expressed in normal kidney mesangial cells, and it translocates from the cytosol to the membrane using RhoA^+^ vesicles. The inhibitor of vesicle trafficking pathway, Exo1, significantly reduces the transport of TGase2 to the membrane. (**B**) Polymeric IgA1 (pIgA1) from the blood in patients with IgA nephropathy enter into the kidney and can be deposited in the mesangium through binding to TGase2 and the IgA1 receptor TFRC; this activates more expression of TGase2, RhoA, and RhoA^+^ vesicles. Increased TGase2 are transported to the membrane through RhoA^+^ vesicles, leading more and more pIgA1 sticks to the mesangial cell membrane. (**C**) The interaction of pIgA1 with mesangial cells induces cell proliferation and production of ECM, such as fibronectin and collagens, which will further increase the disease progression.

In conclusion, we have identified the molecular mechanism of TGase2 in regulating mesangial pIgA1 deposition in IgAN (Figure 7). TGase2 is weakly expressed in normal kidney mesangial cells, and it translocates from the cytosol to the membrane using RhoA^+^ vesicles. Polymeric IgA1 comes from the blood into the kidney and induces an increase of TGase2 expression as well as RhoA^+^ vesicles in the mesangial cells. The increased numbers of TGase2 are transferred to the membrane with the help of RhoA^+^ vesicles, leading to more and more pIgA1 sticking to the mesangial cell membrane, thanks to the interaction of pIgA1 with IgA1 receptor (TFRC) and TGase2. Massive pIgA1 deposition causes abnormal mesangial cell proliferation, ECM deposition, and thus kidney fibrosis. Our study suggests that pharmacologic modulation of vesicular TGase2 could impede transfer of TGase2 from cytosol to membrane and reduce pIgA1 deposition on mesangial cells, which could be of interest as a treatment strategy for IgAN.

## Methods

### Polymeric IgA1 preparation.

Patients with primary IgAN confirmed by renal biopsy were included. Exclusion criteria of patients with IgAN included secondary IgAN, such as systemic lupus erythematosus, Henoch-Schonlein purpura, and hepatic diseases; current or recent (within 90 days) exposure to corticosteroids; or immunosuppressive therapy. Blood samples of patients with IgAN were obtained the day of renal biopsy. Healthy donors with normal urinalysis and without history of kidney disease and medication use, such as corticosteroids and immunosuppressive drugs, were recruited as normal controls in the physical examination center. Blood samples of normal controls were obtained at the day of physical examination. Serum was isolated via centrifuge and frozen in liquid at –80°C.

As previously described (45), IgA1 in sera from 20 healthy donors or 20 patients with IgAN was isolated using Jacalin (Vector Laboratories, AL-1153-10) affinity chromatography; then, polymeric IgA1 (pIgA1) and monomeric IgA1 (mIgA1) fractions were separated by Sephacryl S-200 (GE Healthcare, 17-1166-01) molecular sieve chromatography. The clinical data of healthy donors and patients with IgAN were summarized in Supplemental Table 3. Affinity chromatography and molecular sieve chromatography were performed using Biologic DuoFlow medium-pressure chromatography system (Bio-Rad). Polymeric and monomeric IgA1 were identified by native-PAGE with Coomassie brilliant blue staining and Western blot using anti–human IgA1 antibody (Southern Biotech, B3506B4).

### Human mesangial cells culture.

The HMC (gifted by Xiaoyun Jiang, Professor of the Department of Pediatrics at the First Affiliated Hospital of Sun Yat-sen University, Guangzhou) was cultured in RPMI-1640 medium (Thermo Fisher Scientific, 22400-089) containing 10% FBS (Thermo Fisher Scientific, 10091-148), penicillin (100 U/mL), and streptomycin (100 U/mL) at 37°C in a humidified atmosphere of 5% CO_2_. Cells grown at passages 3–8 were used in all experiments.

### Flow cytometric analysis.

HMC were grown to log phase in RPMI 1640 medium with 10% FBS and harvested by trypsin digestion. Cells were adjusted to 1.6 × 10^5^/mL, and 200 μL of cell suspension was used in binding assays. HMC suspension was preincubated with or without TGase2 inhibitor LDN-21279 (MedChemExpress, HY-16693) or exocytosis inhibitor Exo1 (MedChemExpress, HY-112670); it was then incubated with pIgA1 isolated from patients with IgAN. After incubation, cells were used for flow cytometric analysis. In brief, HMC were washed with 0.5% BSA-PBS and then incubated with FITC-conjugated goat anti–human IgA antibody (Origo, ARG21910) or goat anti–human IgA antibody (Origo, ARG21909) and Alexa Fluor 647–conjugated donkey anti-goat secondary antibody (Abcam, ab150131). The stained cells were analyzed on a Becton Dickinson Model FACScan (BD Biosciences). A minimum of 1 × 10^4^ fixed cells for each sample was analyzed. The data were processed by using the FlowJo v10 software (Tree Star Inc.). The gating strategy of flow cytometric analysis was based on the forward and side scatter properties of HMC to remove dead cells that could increase autofluorescence and nonspecific binding of antibodies. 

### Cell survival.

HMC were maintained in RPMI-1640 medium with 10% FBS. To study the effects of TGase2 inhibitor LDN-21279 on cell survival, HMC were seeded into 12-well plates at a density of 1 ***×*** 10^4^ cells/well. After cells were serum starved for 2 hours, various concentrations of LDN-21279 were then added, and cells were grown in serum-free medium for 48 hours. The rates of apoptosis and survival were determined with FCM analysis by making use of an Annexin V-APC/PI Apoptosis Assay kit (UElandy, A6030L) according to the instructions provided by the manufacturer. The reading of results was carried out on a Becton Dickinson Model FACScan. The data were processed using the FlowJo v10 software.

For the cell proliferation assay, HMC were seeded into 96-well plates at a density of 5,000 cells/well. After cells were serum starved for 2 hours, various concentrations of pIgA1 from patients with IgAN were then added, and cells were grown in serum-free medium. Cell survival proliferation during growth periods was measured using the MTS assay (Cell Titer 96 Aqueous Nonradioactive Cell Proliferation Assay; Promega, G109A) following the manufacturer instruction. The plates were incubated for 4 hours at 37°C after addition 20 μL assay solution. Subsequently, the absorbance was measured at 490 nm by an ELISA reader (Molecular Devices).

### Isolation of cytosol complexes and data acquisition by MS.

To isolate TGase2-associated proteins from HMC, HMC monolayers were incubated with or without pIgA1 (10 μg/mL) for 48 hours at 37°C. After washing with PBS, cytosolic proteins were separated from the membrane fraction using ProteoExtract Transmembrane Protein Extraction kit (MilliporeSigma, 71772-3). Cytosolic and membrane enrichment were validated by Western blotting. The crude cytosol fraction was resuspended in IP buffer (Thermo Fisher Scientific, 87787) containing protease inhibitor cocktail (Roche, 04693159001). Then, TGase2-associated proteins were immunoprecipitated using the protein A/G magnetic beads (Santa Cruz Biotechnology Inc., sc-2003), of which rabbit anti-TGase2 antibody (Abcam, ab64771) was crosslinked using disuccinimidyl suberate. Incubation of cytosol lysates with the antibody-coated beads were performed for overnight at 4°C in constant rotation. Rabbit IgG (Abcam, ab172730) IP was included as negative control. TGase2-associated proteins were removed from the beads after washing the beads 5 times with PBS buffer. The immunoprecipitants were analyzed by Western blot. Two independent TGase2 IP experiments were carried out.

Proteins were digested with trypsin (Promega, V5111), and peptides were analyzed by high-performance liquid chromatography–tandem MS (HPLC-MS/MS) using a Q Exactive Mass Spectrometer (Thermo Fisher Scientific). Protein identification was performed with Mascot software by searching Uniprot human protein database. Proteins with fewer than 2 total peptides were removed to exclude technical artifacts. The functional enrichment and pathway analysis of TGase2-pulldown proteins were performed using Gene Ontology (GO) database (http://geneontology.org). PPI network was analyzed using STRING V10.0 (http://stringdb.org).

### Cellular vesicle isolation and characterization.

Cellular vesicles were isolated from HMC by filtration following the instruction from Bestbio (BB-36914). The purified vesicles were resuspended in PBS for nanoparticle tracking analysis (NTA) to examine the microparticle size and distribution. NTA was performed using NanoSight NS300 (Malvern) according to the operating instructions, without any changes. Western blot analysis was also used to verify the nature of the isolated vesicles.

### Plasmid constructs and transfections.

The following constructs were prepared: pcDNA3.1(+)-RhoA, where human RhoA cDNA (NM_001664.3) was subcloned into pcDNA3.1(+). Empty vector was used as a negative control. All constructs were verified by Sanger sequencing. RhoA was knocked down by transient transfection with human RhoA-targeting siRNA (5′-CGAUGUUAUACUGAUGUGUUUTT-3′) or scrambled control siRNA (5′-UUCUCCGAACGUGUCACGUTT-3′). Transient transfection of HMC cells was performed using Lipofectamine 3000 (Invitrogen, L3000-015) reagent according to the manufacture’s instruction.

### Western blot.

Proteins were separated in 4%–20% sodium dodecyl sulfate–polyacrylamide gel electrophoresis in reducing conditions and transferred on polyvinylidene difluoride membranes (MilliporeSigma, ISEQ00010). After blocking with 5% milk, membranes were probed with rabbit anti–human Fn (Epitomics, 1574-1), mouse anti–human collagen type I (Proteintech, 66761-1), rabbit anti–human TFRC (Zen BioScience, 619871), rabbit anti–human TGase2 (Abcam, ab64771), mouse anti–human TGase2 (Abcam, ab2386), rabbit anti–human RhoA (ABclonal, A13947), mouse anti–human RhoA (Santa Cruz Biotechnology Inc., sc-418), mouse anti–human IgA antibody (Southern Biotech, B3506B4), rabbit anti–human GAPDH (Bioworld Technology, AP0063), rabbit anti–human β-tubulin (ZEN Bio, 380628), rabbit anti–human Na^+^/K^+^ ATPase1 (ABclonal, A7878), or rabbit anti–Flotillin 2 (GeneTex, GTX114411) and detected with horseradish peroxidase–conjugated anti-mouse or anti-rabbit antibodies and ECL-Plus detecting system (GE Healthcare).

### Immunofluorescence analysis.

Five cases of renal biopsies of patients with IgAN (Supplemental Table 4) and 3 cases of renal biopsies from kidney donors served as normal controls; they were formalin-fixed and subsequently paraffin embedded. Then, 4 μm serial sections were deparaffinized and rehydrated, and antigen retrieval was performed in 0.01 mol/L sodium citrate buffer, pH 6.0, in an antigen retriever (121°C) for 15 minutes. For immunofluorescence staining of cells, cells were fixed in 4% paraformaldehyde for 10 minutes and then washed with PBS. After blocking with 3% BSA in PBS, tissue sections or fixed cells were incubated overnight at 4°C with mouse anti-TGase2 antibody (Abcam, ab2386) or FITC-conjugated goat anti–human IgA antibody (Origo, ARG21910). Bound antibodies were detected by Alexa 546–conjugated donkey anti–mouse IgG secondary antibody (Invitrogen, A10036). Nuclei were stained with DAPI (MilliporeSigma, MBD0015), and slides were mounted with ProLong Gold Antifade Reagent (Invitrogen, P36934). Immunofluorescence evaluation was performed and analyzed with an LSM 510 Meta confocal laser-scanning microscope (Carl Zeiss). Images from each section were analyzed, and positive signals in a region of interest were quantified using ImageJ software (NIH) following a detailed report (46). Briefly, we used the Hyperstack and Colorized options to analyze each of the fluorescence channels collected in the original experiments. The drawing pen was used to circle the area of the tissue to quantify and measure the mean intensity value.

### Statistics.

All the experiments were performed in 2–3 independent biological or experimental replicates. Statistical analyses were performed with GraphPad Prism version 8. Significant differences were determined by 2-tailed Student’s *t* test, 1-way ANOVA, or 2-way ANOVA. Statistical tests are described in each figure legend. Differences were considered statistically significant at *P* < 0.05.

### Study approval.

All work contained in this publication was approved by the IRB of the First Affiliated Hospital of Sun Yat-sen University (no. [2020]511). All participants gave written informed consent in accordance with the ethics principles stated in the Declaration of Helsinki.

### Data availability.

Values for all data points found in graphs can be found in the Supporting Data Values file, and all the data are available from the corresponding author upon reasonable request.

## Author contributions

SF designed the experiments. The order of co–first authors ZZ and ZL was determined by their contribution to the article. ZZ, ZL, LJ,and SF organized the samples, conducted the experiments, and performed data analysis. YL, QK, and WC provided experimental and technical support. ZZ, ZL, and SF prepared the manuscript. ZZ and SF edited and revised the manuscript.

## Supplementary Material

Supplemental data

Supporting data values

## Version 1 10/09/2023

Electronic publication
