# Supplementary material for: RhoA vesicle trafficking–mediated transglutaminase 2 membrane translocation promotes IgA1 mesangial deposition in IgA nephropathy
Source: JCI Insight. 2023 Oct 9;8(19):e160374. doi: 10.1172/jci.insight.160374 (PMC10619437; doi:10.1172/jci.insight.160374)
Supplement: Supplemental data [file jciinsight-8-160374-s160.pdf]

Table S1. TGase2 partner proteins in the cytosolic fraction of HMC without pIgA1 treatment

Uniprot/ Swissprot no.	Identification	Name	Score	Matches	Number of peptides
O43707	Alpha-actinin-4	ACTN4	1714	59	33
P35579	Myosin-9	MYH9	1660	57	43
Q15149	Plectin	PLEC	1501	61	58
P08238	Heat shock protein HSP 90-beta	HS90B	1489	57	27
P68371	Tubulin beta-4B chain	TBB4B	1449	56	15
P07900	Heat shock protein HSP 90-alpha	HS90A	1434	61	30
P07437	Tubulin beta chain	TBB5	1408	53	15
P12814	Alpha-actinin-1	ACTN1	1356	53	31
P60709	Actin, cytoplasmic 1	ACTB	1353	58	13
P46940	Ras GTPase-activating-like protein IQGAP1	IQGA1	1288	53	39
P14618	Pyruvate kinase PKM	KPYM	1242	47	22
P49327	Fatty acid synthase	FAS	1131	49	40
P68363	Tubulin alpha-1B chain	TBA1B	984	34	12
Q00610	Clathrin heavy chain 1	CLH1	959	35	29
O75369	Filamin-B	FLNB	947	37	33
Q14204	Cytoplasmic dynein 1 heavy chain 1	DYHC1	912	36	36
Q9BQE3	Tubulin alpha-1C chain	TBA1C	812	30	12
Q9Y490	Talin-1	TLN1	788	26	22
P21333	Filamin-A	FLNA	730	31	29
Q9BUF5	Tubulin beta-6 chain	TBB6	672	26	10
P68032	Actin, alpha cardiac muscle 1	ACTC	668	37	8
P13639	Elongation factor 2	EF2	650	29	20
P35580	Myosin-10	MYH10	567	20	14

P07355	Annexin A2	ANXA2	523	13	9
P06733	Alpha-enolase	ENOA	486	13	8
P14625	Endoplasmin	ENPL	482	16	14
P11142	Heat shock cognate 71 kDa protein	HSP7C	441	16	10
Q14974	Importin subunit beta-1	IMB1	414	12	10
P50990	T-complex protein 1 subunit theta	TCPQ	400	16	13
P20591	Interferon-induced GTP-binding protein Mx1	MX1	399	17	13
P46060	Ran GTPase-activating protein 1	RAGP1	380	10	10
Q9P2E9	Ribosome-binding protein 1	RRBP1	373	11	8
Q92973	Transportin-1	TNPO1	372	12	9
Q7Z6Z7	E3 ubiquitin-protein ligase HUWE1	HUWE1	362	15	14
O60763	General vesicular transport factor p115	USO1	360	10	9
P78371	T-complex protein 1 subunit beta	TCPB	357	14	12
Q93008	Probable ubiquitin carboxyl-terminal hydrolase FAF-X	USP9X	342	12	12
P26641	Elongation factor 1-gamma	EF1G	324	12	10
P50991	T-complex protein 1 subunit delta	TCPD	312	8	6
Q9Y230	RuvB-like 2	RUVB2	307	10	10
P11586	C-1-tetrahydrofolate synthase, cytoplasmic	C1TC	293	11	10
P27348	14-3-3 protein theta	1433T	275	8	4
P30153	Serine/threonine-protein phosphatase 2A 65 kDa regulatory subunit A alpha isoform	2AAA	262	10	8
P13797	Plastin-3	PLST	258	7	6
P62258	14-3-3 protein epsilon	1433E	251	8	4
P63104	14-3-3 protein zeta/delta	1433Z	251	7	3
P55072	Transitional endoplasmic reticulum ATPase	TERA	249	10	8
Q15084	Protein disulfide-isomerase A6	PDIA6	245	6	6

Q99832	T-complex protein 1 subunit eta	TCPH	242	12	11
P61981	14-3-3 protein gamma	1433G	233	7	3
P22234	Multifunctional protein ADE2	PUR6	228	6	4
Q27J81	Inverted formin-2	INF2	225	6	6
P09914	Interferon-induced protein with tetratricopeptide repeats 1	IFIT1	218	10	7
P04632	Calpain small subunit 1	CPNS1	216	5	3
P53396	ATP-citrate synthase	ACLY	212	9	7
O14879	Interferon-induced protein with tetratricopeptide repeats 3	IFIT3	207	9	6
Q8WUM4	Programmed cell death 6-interacting protein	PDC6I	206	8	8
P05198	Eukaryotic translation initiation factor 2 subunit 1	IF2A	205	8	8
P13489	Ribonuclease inhibitor	RINI	205	5	5
P18085	ADP-ribosylation factor 4	ARF4	200	7	4
P31946	14-3-3 protein beta/alpha	1433B	199	6	2
P53621	Coatomer subunit alpha	COPA	198	10	10
Q01813	ATP-dependent 6-phosphofructokinase, platelet type	PFKAP	196	9	7
P04083	Annexin A1	ANXA1	192	4	4
P60660	Myosin light polypeptide 6	MYL6	189	7	6
P61289	Proteasome activator complex subunit 3	PSME3	188	7	6
P17858	ATP-dependent 6-phosphofructokinase, liver type	PFKAL	174	7	6
Q9Y265	RuvB-like 1	RUVB1	173	9	8
Q07812	Apoptosis regulator BAX	BAX	172	3	3
P53618	Coatomer subunit beta	COPB	170	8	8
Q15366	Poly(rC)-binding protein 2	PCBP2	167	4	4
Q9Y678	Coatomer subunit gamma-1	COPG1	164	7	7
Q15365	Poly(rC)-binding protein 1	PCBP1	162	4	4
O60701	UDP-glucose 6-dehydrogenase	UGDH	159	6	6

Q01518	Adenylyl cyclase-associated protein 1	CAP1	159	9	7
P07237	Protein disulfide-isomerase	PDIA1	158	5	5
P11021	Endoplasmic reticulum chaperone BiP	BIP	155	5	4
P10321	HLA class I histocompatibility antigen, C alpha chain	HLAC	154	4	4
P32119	Peroxiredoxin-2	PRDX2	153	6	5
O43175	D-3-phosphoglycerate dehydrogenase	SERA	150	4	4
P40227	T-complex protein 1 subunit zeta	TCPZ	146	8	7
P61923	Coatomer subunit zeta-1	COPZ1	146	4	3
P18669	Phosphoglycerate mutase 1	PGAM1	143	7	5
O95340	Bifunctional 3~-phosphoadenosine 5~-phosphosulfate synthase 2	PAPS2	142	5	3
P04406	Glyceraldehyde-3-phosphate dehydrogenase	G3P	140	5	4
P51149	Ras-related protein Rab-7a	RAB7A	135	4	3
P54920	Alpha-soluble NSF attachment protein	SNAA	129	4	4
O75340	Programmed cell death protein 6	PDCD6	126	4	4
O00429	Dynamin-1-like protein	DNM1L	125	4	4
P17987	T-complex protein 1 subunit alpha	TCPA	124	7	6
P35606	Coatomer subunit beta~	COPB2	118	7	5
Q12905	Interleukin enhancer-binding factor 2	ILF2	118	5	4
P07099	Epoxide hydrolase 1	HYEP	117	4	4
Q06830	Peroxiredoxin-1	PRDX1	115	5	5
P50995	Annexin A11	ANX11	113	4	4
P08195	4F2 cell-surface antigen heavy chain	4F2	112	5	5
Q9NYL9	Tropomodulin-3	TMOD3	110	2	2
P33176	Kinesin-1 heavy chain	KINH	108	4	4
P06493	Cyclin-dependent kinase 1	CDK1	107	3	3
Q9UL46	Proteasome activator complex subunit 2	PSME2	107	4	3

P20073	Annexin A7	ANXA7	106	3	3
O76094	Signal recognition particle subunit SRP72	SRP72	105	3	3
P37108	Signal recognition particle 14 kDa protein	SRP14	104	1	1
Q96TA1	Protein Niban 2	NIBA2	102	6	6
O43399	Tumor protein D54	TPD54	98	3	3
Q99873	Protein arginine N-methyltransferase 1	ANM1	97	2	2
Q9H223	EH domain-containing protein 4	EHD4	97	2	2
P48643	T-complex protein 1 subunit epsilon	TCPE	95	6	6
P11413	Glucose-6-phosphate 1-dehydrogenase	G6PD	91	2	2
P30101	Protein disulfide-isomerase A3	PDIA3	91	3	3
P12268	Inosine-5~-monophosphate dehydrogenase 2	IMDH2	90	3	3
P51148	Ras-related protein Rab-5C	RAB5C	90	4	4
Q15907	Ras-related protein Rab-11B	RB11B	90	2	2
Q07960	Rho GTPase-activating protein 1	RHG01	89	3	3
Q92616	eIF-2-alpha kinase activator GCN1	GCN1	88	5	5
Q9NZN4	EH domain-containing protein 2	EHD2	87	3	3
Q14008	Cytoskeleton-associated protein 5	CKAP5	85	2	2
O15212	Prefoldin subunit 6	PFD6	84	1	1
Q92598	Heat shock protein 105 kDa	HS105	84	3	3
Q9NR31	GTP-binding protein SAR1a	SAR1A	83	2	2
Q9NT62	Ubiquitin-like-conjugating enzyme ATG3	ATG3	82	2	2
O00170	AH receptor-interacting protein	AIP	80	3	3
P00338	L-lactate dehydrogenase A chain	LDHA	80	2	2
P61026	Ras-related protein Rab-10	RAB10	76	3	3
Q9Y6B6	GTP-binding protein SAR1b	SAR1B	74	3	2
Q92597	Protein NDRG1	NDRG1	74	1	1

P17655	Calpain-2 catalytic subunit	CAN2	70	3	3
P78344	Eukaryotic translation initiation factor 4 gamma 2	IF4G2	70	2	2
Q9H0U4	Ras-related protein Rab-1B	RAB1B	70	3	3
Q15691	Microtubule-associated protein RP/EB family member 1	MARE1	69	2	1
P05187	Alkaline phosphatase, placental type	PPB1	69	2	2
P40121	Macrophage-capping protein	CAPG	68	2	2
O95747	Serine/threonine-protein kinase	OXSR1	67	1	1
Q9UNH7	Sorting nexin-6	SNX6	67	3	2
Q9Y371	Endophilin-B1	SHLB1	67	1	1
P00558	Phosphoglycerate kinase 1	PGK1	66	3	3
O00203	AP-3 complex subunit beta-1	AP3B1	65	2	2
P43490	Nicotinamide phosphoribosyltransferase	NAMPT	65	4	4
O95816	BAG family molecular chaperone regulator 2	BAG2	64	2	2
P04818	Thymidylate synthase	TYSY	64	2	2
P21980	Protein-glutamine gamma-glutamyltransferase 2	TGM2	64	2	1
P57764	Gasdermin-D	GSDMD	63	2	2
O60749	Sorting nexin-2	SNX2	60	1	1
Q8IWZ3	Ankyrin repeat and KH domain-containing protein 1	ANKH1	60	2	2
P63208	S-phase kinase-associated protein 1	SKP1	59	2	2
Q9Y2Z0	Protein SGT1 homolog	SGT1	59	2	2
Q15437	Protein transport protein Sec23B	SC23B	59	3	3
Q00341	Vigilin	VIGLN	58	4	4
Q7Z2W4	Zinc finger CCCH-type antiviral protein 1	ZCCHV	58	1	1
O60493	Sorting nexin-3	SNX3	57	1	1
P14923	Junction plakoglobin	PLAK	57	1	1
P23921	Ribonucleoside-diphosphate reductase large subunit	RIR1	57	2	2

Q9UL25	Ras-related protein Rab-21	RAB21	57	2	2
Q9HCE1	Helicase MOV-10	MOV10	56	2	2
Q9H4M9	EH domain-containing protein 1	EHD1	55	2	2
O95433	Activator of 90 kDa heat shock protein ATPase homolog 1	AHSA1	54	3	3
P28482	Mitogen-activated protein kinase 1	MK01	54	2	2
Q96A49	Synapse-associated protein 1	SYAP1	54	1	1
P22061	Protein-L-isoaspartate(D-aspartate) O-methyltransferase	PIMT	53	2	2
P06753	Tropomyosin alpha-3 chain	TPM3	51	1	1
P08758	Annexin A5	ANXA5	50	2	2
P04843	Dolichyl-diphosphooligosaccharide--protein glycosyltransferase subunit 1	RPN1	47	1	1
P62330	ADP-ribosylation factor 6	ARF6	46	2	1
P06730	Eukaryotic translation initiation factor 4E	IF4E	45	2	2
P47756	F-actin-capping protein subunit beta	CAPZB	45	2	2
P40261	Nicotinamide N-methyltransferase	NNMT	44	3	3
Q14697	Neutral alpha-glucosidase AB	GANAB	44	1	1
O14617	AP-3 complex subunit delta-1	AP3D1	42	1	1
P07737	Profilin-1	PROF1	42	1	1
O00151	PDZ and LIM domain protein 1	PDLI1	41	1	1
O95721	Synaptosomal-associated protein 29	SNP29	40	1	1
Q9H074	Polyadenylate-binding protein-interacting protein 1	PAIP1	40	1	1
P40616	ADP-ribosylation factor-like protein 1	ARL1	40	2	2
P53365	Arfaptin-2	ARFP2	39	1	1
P61586	Transforming protein RhoA	RHOA	39	5	3
P13861	cAMP-dependent protein kinase type II-alpha regulatory subunit	KAP2	38	1	1
Q8IYD1	Eukaryotic peptide chain release factor GTP-binding subunit ERF3B	ERF3B	38	1	1

P31948	Stress-induced-phosphoprotein 1	STIP1	37	2	2
P36952	Serpin B5	SPB5	36	1	1
P60903	Protein S100-A10	S10AA	36	1	1
Q01650	Large neutral amino acids transporter small subunit 1	LAT1	36	1	1
P40855	Peroxisomal biogenesis factor 19	PEX19	35	1	1
Q14134	Tripartite motif-containing protein 29	TRI29	35	1	1
P60953	Cell division control protein 42 homolog	CDC42	34	1	1
Q99614	Tetratricopeptide repeat protein 1	TTC1	34	1	1
Q9BRQ8	Ferroptosis suppressor protein 1	FSP1	34	1	1
Q9Y5A9	YTH domain-containing family protein 2	YTHD2	34	1	1
P08134	Rho-related GTP-binding protein RhoC	RHOC	33	1	1
Q9NUQ8	ATP-binding cassette sub-family F member 3	ABCF3	33	1	1
Q8NCA5	Protein FAM98A	FA98A	33	1	1
P02786	Transferrin receptor protein 1	TFR1	33	2	2
Q15181	Inorganic pyrophosphatase	IPYR	32	1	1
Q9UQB8	Brain-specific angiogenesis inhibitor 1-associated protein 2	BAIP2	32	1	1
O95782	AP-2 complex subunit alpha-1	AP2A1	31	2	2
Q13283	Ras GTPase-activating protein-binding protein 1	G3BP1	31	1	1
Q96C19	EF-hand domain-containing protein D2	EFHD2	30	1	1
Q99816	Tumor susceptibility gene 101 protein	TS101	29	1	1
P63244	Receptor of activated protein C kinase 1	RACK1	29	1	1
P36873	Serine/threonine-protein phosphatase PP1-gamma catalytic subunit	PP1G	28	1	1
P49458	Signal recognition particle 9 kDa protein	SRP09	27	1	1
O95486	Protein transport protein Sec24A	SC24A	26	1	1
P55735	Protein SEC13 homolog	SEC13	26	1	1
Q16555	Dihydropyrimidinase-related protein 2	DPYL2	25	1	1

Q9H6S3	Epidermal growth factor receptor kinase substrate 8-like protein 2	ES8L2	25	1	1
P50851	Lipopolysaccharide-responsive and beige-like anchor protein	LRBA	25	1	1
P37802	Transgelin-2	TAGL2	23	1	1
Q92945	Far upstream element-binding protein 2	FUBP2	22	1	1
P52907	F-actin-capping protein subunit alpha-1	CAZA1	20	1	1

Table S2. TGase2 partner proteins in the cytosolic fraction of HMC with pIgA1 treatment

Uniprot/Swissprot no.	Identification	Name	Score	Matches	Number of peptides
P07437	Tubulin beta chain	TBB5	1882	76	16
P68371	Tubulin beta-4B chain	TBB4B	1800	75	15
P60709	Actin, cytoplasmic 1	ACTB	1353	51	15
P46940	Ras GTPase-activating-like protein IQGAP1	IQGA1	1202	41	35
P68363	Tubulin alpha-1B chain	TBA1B	1079	43	15
Q9BQE3	Tubulin alpha-1C chain	TBA1C	1008	40	14
P14618	Pyruvate kinase PKM	KPYM	996	40	21
P08238	Heat shock protein HSP 90-beta	HS90B	948	38	24
P68032	Actin, alpha cardiac muscle 1	ACTC	844	41	11
Q9BUF5	Tubulin beta-6 chain	TBB6	806	32	8
P07900	Heat shock protein HSP 90-alpha	HS90A	767	28	20
P35579	Myosin-9	MYH9	740	29	24
P11142	Heat shock cognate 71 kDa protein	HSP7C	684	26	19
O43707	Alpha-actinin-4	ACTN4	658	23	20
Q15149	Plectin	PLEC	583	26	26
P13639	Elongation factor 2	EF2	579	23	15
P12814	Alpha-actinin-1	ACTN1	460	21	18
Q14204	Cytoplasmic dynein 1 heavy chain 1	DYHC1	447	16	16
P07355	Annexin A2	ANXA2	446	17	11
P46060	Ran GTPase-activating protein 1	RAGP1	424	12	10
P22234	Multifunctional protein ADE2	PUR6	407	12	10
Q9Y230	RuvB-like 2	RUVB2	401	13	12
P11021	Endoplasmic reticulum chaperone BiP	BIP	389	12	11

P49327	Fatty acid synthase	FAS	387	19	18
Q9P2E9	Ribosome-binding protein 1	RRBP1	382	11	8
P06733	Alpha-enolase	ENOA	317	11	10
P20591	Interferon-induced GTP-binding protein Mx1	MX1	301	15	12
P04632	Calpain small subunit 1	CPNS1	282	7	5
P14625	Endoplasmin	ENPL	269	8	8
P26641	Elongation factor 1-gamma	EF1G	262	12	10
Q15365	Poly(rC)-binding protein 1	PCBP1	259	8	6
Q96A49	Synapse-associated protein 1	SYAP1	258	8	6
P13489	Ribonuclease inhibitor	RINI	256	5	5
Q01813	ATP-dependent 6-phosphofructokinase, platelet type	PFKAP	252	8	6
Q9Y265	RuvB-like 1	RUVB1	250	11	9
P50990	T-complex protein 1 subunit theta	TCPQ	249	10	10
P50991	T-complex protein 1 subunit delta	TCPD	244	10	9
Q99832	T-complex protein 1 subunit eta	TCPH	244	11	10
Q15366	Poly(rC)-binding protein 2	PCBP2	242	9	7
Q16555	Dihydropyrimidinase-related protein 2	DPYL2	242	10	9
Q7Z6Z7	E3 ubiquitin-protein ligase HUWE1	HUWE1	238	9	9
P08195	4F2 cell-surface antigen heavy chain	4F2	233	7	7
Q9Y490	Talin-1	TLN1	227	12	12
O60763	General vesicular transport factor p115	USO1	224	5	5
P18085	ADP-ribosylation factor 4	ARF4	224	10	5
P21333	Filamin-A	FLNA	222	10	10
P04083	Annexin A1	ANXA1	220	5	5
Q15084	Protein disulfide-isomerase A6	PDIA6	219	4	4
P62937	Peptidyl-prolyl cis-trans isomerase A	PPIA	213	7	5

P04439	HLA class I histocompatibility antigen, A alpha chain	HLAA	213	6	5
Q27J81	Inverted formin-2	INF2	213	7	6
P17858	ATP-dependent 6-phosphofructokinase, liver type	PFKAL	212	6	5
P35580	Myosin-10	MYH10	210	9	6
Q9NZN4	EH domain-containing protein 2	EHD2	206	10	9
P35606	Coatomer subunit beta~	COPB2	204	9	9
P09914	Interferon-induced protein with tetratricopeptide repeats 1	IFIT1	196	5	5
P04406	Glyceraldehyde-3-phosphate dehydrogenase	G3P	188	8	7
O43399	Tumor protein D54	TPD54	186	4	4
P53621	Coatomer subunit alpha	COPA	178	10	10
Q99873	Protein arginine N-methyltransferase 1	ANM1	177	5	5
P21980	Protein-glutamine gamma-glutamyltransferase 2	TGM2	169	7	7
O43175	D-3-phosphoglycerate dehydrogenase	SERA	165	5	5
Q8WUM4	Programmed cell death 6-interacting protein	PDC6I	165	6	6
O76094	Signal recognition particle subunit SRP72	SRP72	164	4	4
P30101	Protein disulfide-isomerase A3	PDIA3	162	8	8
P43490	Nicotinamide phosphoribosyltransferase	NAMPT	162	11	10
P06493	Cyclin-dependent kinase 1	CDK1	161	6	5
P11586	C-1-tetrahydrofolate synthase, cytoplasmic	C1TC	161	6	6
P05198	Eukaryotic translation initiation factor 2 subunit 1	IF2A	157	7	6
P78371	T-complex protein 1 subunit beta	TCPB	156	6	5
P54920	Alpha-soluble NSF attachment protein	SNAA	155	6	6
P40227	T-complex protein 1 subunit zeta	TCPZ	154	8	8
P63244	Receptor of activated protein C kinase 1	RACK1	152	6	5
Q00610	Clathrin heavy chain 1	CLH1	152	4	4
Q13596	Sorting nexin-1	SNX1	151	5	4

P78344	Eukaryotic translation initiation factor 4 gamma 2	IF4G2	149	9	8
P61981	14-3-3 protein gamma	1433G	149	6	4
P31946	14-3-3 protein beta/alpha	1433B	148	6	4
Q9Y2Z0	Protein SGT1 homolog	SGT1	146	5	5
O60664	Perilipin-3	PLIN3	146	5	4
P47756	F-actin-capping protein subunit beta	CAPZB	145	5	5
P07237	Protein disulfide-isomerase	PDIA1	144	5	4
Q9Y6B6	GTP-binding protein SAR1b	SAR1B	139	4	3
P62258	14-3-3 protein epsilon	1433E	135	6	4
Q9Y678	Coatomer subunit gamma-1	COPG1	134	4	4
P27348	14-3-3 protein theta	1433T	134	5	3
Q07960	Rho GTPase-activating protein 1	RHG01	133	5	5
Q96TA1	Protein Niban 2	NIBA2	132	4	4
P07737	Profilin-1	PROF1	131	4	4
Q14697	Neutral alpha-glucosidase AB	GANAB	130	5	5
P63104	14-3-3 protein zeta/delta	1433Z	128	5	3
Q06830	Peroxiredoxin-1	PRDX1	127	10	7
Q9UL25	Ras-related protein Rab-21	RAB21	124	2	2
P53618	Coatomer subunit beta	COPB	122	6	6
O14879	Interferon-induced protein with tetratricopeptide repeats 3	IFIT3	120	6	6
O00429	Dynamin-1-like protein	DNM1L	120	5	5
Q9H4M9	EH domain-containing protein 1	EHD1	120	5	4
P51149	Ras-related protein Rab-7a	RAB7A	119	3	2
Q14134	Tripartite motif-containing protein 29	TRI29	117	6	5
P00558	Phosphoglycerate kinase 1	PGK1	112	4	4
Q14008	Cytoskeleton-associated protein 5	CKAP5	109	2	2

P50995	Annexin A11	ANX11	108	5	5
P60660	Myosin light polypeptide 6	MYL6	107	5	4
Q9NR31	GTP-binding protein SAR1a	SAR1A	107	2	2
Q99614	Tetratricopeptide repeat protein 1	TTC1	106	5	4
Q9H0U4	Ras-related protein Rab-1B	RAB1B	106	4	4
P31948	Stress-induced-phosphoprotein 1	STIP1	106	4	4
Q9H223	EH domain-containing protein 4	EHD4	104	3	3
P15311	Ezrin	EZRI	104	6	6
P08134	Rho-related GTP-binding protein RhoC	RHOC	102	4	3
Q14974	Importin subunit beta-1	IMB1	102	5	4
P00338	L-lactate dehydrogenase A chain	LDHA	102	4	4
O43633	Charged multivesicular body protein 2a	CHM2A	102	2	2
P12268	Inosine-5~-monophosphate dehydrogenase 2	IMDH2	101	3	3
P57764	Gasdermin-D	GSDMD	98	2	2
Q9BRQ8	Ferroptosis suppressor protein 1	FSP1	98	3	3
P33176	Kinesin-1 heavy chain	KINH	97	5	5
Q00341	Vigilin	VIGLN	92	3	3
P17655	Calpain-2 catalytic subunit	CAN2	91	4	4
Q7Z2W4	Zinc finger CCCH-type antiviral protein 1	ZCCHV	91	3	3
Q92616	eIF-2-alpha kinase activator GCN1	GCN1	90	4	4
P40616	ADP-ribosylation factor-like protein 1	ARL1	89	3	2
O75340	Programmed cell death protein 6	PDCD6	89	3	3
P62879	Guanine nucleotide-binding protein G(I)/G(S)/G(T) subunit beta-2	GBB2	87	6	6
O43237	Cytoplasmic dynein 1 light intermediate chain 2	DC1L2	86	2	2
O15212	Prefoldin subunit 6	PFD6	84	1	1
P37802	Transgelin-2	TAGL2	84	2	2

P07099	Epoxide hydrolase 1	HYEP	82	3	2
Q15691	Microtubule-associated protein RP/EB family member 1	MARE1	82	3	2
P52907	F-actin-capping protein subunit alpha-1	CAZA1	80	3	3
P05187	Alkaline phosphatase, placental type	PPB1	79	2	2
Q92973	Transportin-1	TNPO1	79	2	2
O95782	AP-2 complex subunit alpha-1	AP2A1	78	3	3
Q07812	Apoptosis regulator BAX	BAX	78	2	2
P41567	Eukaryotic translation initiation factor 1	EIF1	78	1	1
Q92597	Protein NDRG1	NDRG1	78	4	3
Q15907	Ras-related protein Rab-11B	RB11B	78	3	3
Q9H074	Polyadenylate-binding protein-interacting protein 1	PAIP1	77	3	3
P61289	Proteasome activator complex subunit 3	PSME3	76	3	3
O60493	Sorting nexin-3	SNX3	75	2	2
P31350	Ribonucleoside-diphosphate reductase subunit M2	RIR2	75	3	3
Q92598	Heat shock protein 105 kDa	HS105	74	1	1
P23284	Peptidyl-prolyl cis-trans isomerase B	PPIB	73	3	3
Q8IYD1	Eukaryotic peptide chain release factor GTP-binding subunit ERF3B	ERF3B	73	2	2
P28066	Proteasome subunit alpha type-5	PSA5	73	2	2
P61160	Actin-related protein 2	ARP2	68	3	3
P51148	Ras-related protein Rab-5C	RAB5C	67	3	3
Q93008	Probable ubiquitin carboxyl-terminal hydrolase FAF-X	USP9X	66	4	4
O95747	Serine/threonine-protein kinase OSR1	OXSR1	66	1	1
P49458	Signal recognition particle 9 kDa protein	SRP09	64	1	1
P00568	Adenylate kinase isoenzyme 1	KAD1	64	2	2
Q9UL46	Proteasome activator complex subunit 2	PSME2	63	3	3
P23921	Ribonucleoside-diphosphate reductase large subunit	RIR1	62	3	3

P17987	T-complex protein 1 subunit alpha	TCPA	62	4	3
P48643	T-complex protein 1 subunit epsilon	TCPE	61	2	2
P42771	Cyclin-dependent kinase inhibitor 2A	CDN2A	61	1	1
Q99816	Tumor susceptibility gene 101 protein	TS101	59	3	3
O60701	UDP-glucose 6-dehydrogenase	UGDH	59	3	3
Q9NT62	Ubiquitin-like-conjugating enzyme ATG3	ATG3	58	2	2
O95340	Bifunctional 3~-phosphoadenosine 5~-phosphosulfate synthase 2	PAPS2	57	3	3
P28482	Mitogen-activated protein kinase 1	MK01	56	1	1
P32119	Peroxiredoxin-2	PRDX2	56	2	2
Q99497	Parkinson disease protein 7	PARK7	55	2	2
P22061	Protein-L-isoaspartate(D-aspartate) O-methyltransferase	PIMT	55	2	2
P13861	cAMP-dependent protein kinase type II-alpha regulatory subunit	KAP2	54	2	2
P18669	Phosphoglycerate mutase 1	PGAM1	54	4	4
Q13232	Nucleoside diphosphate kinase 3	NDK3	54	1	1
Q92572	AP-3 complex subunit sigma-1	AP3S1	53	1	1
P39656	Dolichyl-diphosphooligosaccharide--protein glycosyltransferase 48 kDa subunit	OST48	53	1	1
P53365	Arfaptin-2	ARFP2	53	2	2
Q15181	Inorganic pyrophosphatase	IPYR	53	2	2
Q9NRV9	Heme-binding protein 1	HEBP1	52	1	1
Q01650	Large neutral amino acids transporter small subunit 1	LAT1	51	1	1
P04818	Thymidylate synthase	TYSY	51	2	2
P04843	Dolichyl-diphosphooligosaccharide--protein glycosyltransferase subunit 1	RPN1	51	2	2
P08758	Annexin A5	ANXA5	50	2	2
Q9UNH7	Sorting nexin-6	SNX6	50	2	2
P61011	Signal recognition particle 54 kDa protein	SRP54	50	3	3

Q9H6S3	Epidermal growth factor receptor kinase substrate 8-like protein 2	ES8L2	49	1	1
Q3MHD2	Protein LSM12 homolog	LSM12	48	1	1
Q96C19	EF-hand domain-containing protein D2	EFHD2	48	3	3
Q8N5M4	Tetratricopeptide repeat protein 9C	TTC9C	48	2	2
Q13561	Dynactin subunit 2	DCTN2	48	2	2
P61923	Coatomer subunit zeta-1	COPZ1	47	2	2
P61224	Ras-related protein Rap-1b	RAP1B	47	3	3
Q9UN37	Vacuolar protein sorting-associated protein 4A	VPS4A	47	2	2
O95433	Activator of 90 kDa heat shock protein ATPase homolog 1	AHSA1	47	4	4
Q8IYB5	Stromal membrane-associated protein 1	SMAP1	47	1	1
P81605	Dermcidin	DCD	47	1	1
Q9HAD4	WD repeat-containing protein 41	WDR41	47	1	1
Q9NUQ8	ATP-binding cassette sub-family F member 3	ABCF3	46	1	1
P11413	Glucose-6-phosphate 1-dehydrogenase	G6PD	46	1	1
O00170	AH receptor-interacting protein	AIP	46	2	2
Q9Y5A9	YTH domain-containing family protein 2	YTHD2	45	2	2
Q8ND24	RING finger protein 214	RN214	44	1	1
Q9BUL8	Programmed cell death protein 10	PDC10	44	2	2
Q8IWZ3	Ankyrin repeat and KH domain-containing protein 1	ANKH1	44	1	1
O43324	Eukaryotic translation elongation factor 1 epsilon-1	MCA3	43	2	2
Q9HCE1	Helicase MOV-10	MOV10	42	2	2
P40121	Macrophage-capping protein	CAPG	41	2	2
Q9NR46	Endophilin-B2	SHLB2	40	1	1
Q9Y478	5~-AMP-activated protein kinase subunit beta-1	AAKB1	40	1	1
P61586	Transforming protein RhoA	RHOA	39	5	3
P04080	Cystatin-B	CYTB	38	1	1

P37108	Signal recognition particle 14 kDa protein	SRP14	38	1	1
P60903	Protein S100-A10	S10AA	38	2	1
P63208	S-phase kinase-associated protein 1	SKP1	37	1	1
P40855	Peroxisomal biogenesis factor 19	PEX19	37	1	1
P30153	Serine/threonine-protein phosphatase 2A 65 kDa regulatory subunit A alpha isoform	2AAA	37	1	1
Q92945	Far upstream element-binding protein 2	FUBP2	37	2	2
Q13347	Eukaryotic translation initiation factor 3 subunit I	EIF3I	36	1	1
O95816	BAG family molecular chaperone regulator 2	BAG2	35	1	1
P25786	Proteasome subunit alpha type-1	PSA1	35	1	1
P54619	5~-AMP-activated protein kinase subunit gamma-1	AAKG1	35	1	1
Q14671	Pumilio homolog 1	PUM1	35	2	2
P20073	Annexin A7	ANXA7	34	2	2
P11441	Ubiquitin-like protein 4A	UBL4A	34	1	1
Q9UHD1	Cysteine and histidine-rich domain-containing protein 1	CHRD1	34	1	1
Q9H0E2	Toll-interacting protein	TOLIP	34	1	1
Q8NCA5	Protein FAM98A	FA98A	33	1	1
Q9UQB8	Brain-specific angiogenesis inhibitor 1-associated protein 2	BAIP2	33	2	2
P25815	Protein S100-P	S100P	33	1	1
Q12905	Interleukin enhancer-binding factor 2	ILF2	32	1	1
P61962	DDB1- and CUL4-associated factor 7	DCAF7	31	1	1
Q9BY43	Charged multivesicular body protein 4a	CHM4A	31	1	1
Q14247	Src substrate cortactin	SRC8	30	1	1
O14617	AP-3 complex subunit delta-1	AP3D1	30	1	1
P40261	Nicotinamide N-methyltransferase	NNMT	29	2	2
O00203	AP-3 complex subunit beta-1	AP3B1	29	1	1

P53367	Arfaptin-1	ARFP1	28	1	1
Q01518	Adenylyl cyclase-associated protein 1	CAP1	27	1	1
Q9Y263	Phospholipase A-2-activating protein	PLAP	27	1	1
Q13045	Protein flightless-1 homolog	FLII	27	1	1
Q15185	Prostaglandin E synthase 3	TEBP	27	1	1
P55735	Protein SEC13 homolog	SEC13	27	1	1
P16298	Serine/threonine-protein phosphatase 2B catalytic subunit beta isoform	PP2BB	27	1	1
P78318	Immunoglobulin-binding protein 1	IGBP1	26	1	1
Q9UBD9	Cardiotrophin-like cytokine factor 1	CLCF1	25	1	1
Q15020	Squamous cell carcinoma antigen recognized by T-cells 3	SART3	25	1	1
P25788	Proteasome subunit alpha type-3	PSA3	25	1	1
Q96H79	Zinc finger CCCH-type antiviral protein 1-like	ZCCHL	25	1	1
O60841	Eukaryotic translation initiation factor 5B	IF2P	24	1	1
Q9BYX4	Interferon-induced helicase C domain-containing protein 1	IFIH1	24	1	1
P27824	Calnexin	CALX	24	1	1
P14314	Glucosidase 2 subunit beta	GLU2B	24	1	1
Q9NYL9	Tropomodulin-3	TMOD3	24	1	1
Q96P48	Arf-GAP with Rho-GAP domain, ANK repeat and PH domain-containing protein 1	ARAP1	23	1	1
P08236	Beta-glucuronidase	BGLR	22	1	1
P49755	Transmembrane emp24 domain-containing protein 10	TMEDA	22	1	1
P02786	Transferrin receptor protein 1	TFR1	21	1	1
P63010	AP-2 complex subunit beta	AP2B1	21	1	1
P05026	Sodium/potassium-transporting ATPase subunit beta-1	AT1B1	17	1	1
Q02224	Centromere-associated protein E	CENPE	14	1	1

Table S3. Clinical characteristics of IgAN patients and healthy controls

	Healthy control (n=20)	IgAN patients (n=20)	*P* value
Gender (female/male)	11/9	10/10	0.752
Mean age (years)	39.85 ± 7.31	35.45 ± 8.95	0.097
eGFR (ml/min/1.73m^2)	104.62 (88.18, 117.86)	55.72 (36.83, 102.25)	0.008
Serum creatinine (μmol/L)	67.5 (56.5, 80.75)	119.5 (68.5, 161.5)	0.032
Proteinuria range (g/24 h)	0	1.17 (0.56, 2.17)	--
Recurrent hematuria (%)	0	18 (90 %)	--
Oxford classification (%)			
M score			
M0	--	2 (10 %)	
M1	--	18 (90 %)	
E score			
E0	--	17 (85 %)	
E1	--	3 (15 %)	
S score			
S0	--	7 (35 %)	
S1	--	13 (65 %)	
T score			
T0	--	10 (50 %)	
T1	--	8 (40 %)	
T2	--	2 (10 %)	
C score			
C0	--	14 (70 %)	
C1	--	6 (30 %)	
C2	--	0 (0 %)	

Note: eGFR (ml/min/1.73m^2) = 175 × (creatinine, mg/dL)$^{-1.234}$ × (age, years) $^{-0.179}$× (if female, ×0.79). Values are calculated as mean ± SD, median ((interquartile range) or number of patients (%, ratio of group). Baseline characteristics were compared among two groups, using Student's *t*-test, Mann-Whitney *U*-test, or chi-square test as appropriate

Abbreviation: eGFR, estimated glomerular filtration rate; M, Mesangial hypercellularity; E, Endocapillary hypercellularity; S, Segmental glomerulosclerosis; T, Tubular atrophy/interstitial fibrosis; C, Crescents.

Table S4. Clinical-pathological parameters of IgAN patients at the time of renal biopsy used for immunofluorescence analysis

IgAN patients	Age (years)	Sex	eGFR (ml/min/1.73 m^2)	Serum creatine (μmol/L)	Proteinuria (g/24 h)	Glomerulosclerosis	Oxford classification				
							M	S	E	T	C
1	23	female	114.21	60	1.431	1/22 (4.55 %)	M1	S0	E1	T0	C1
2	35	male	64.135	119	2.322	2/21 (9.52 %)	M1	S0	E0	T0	C1
3	65	female	66.364	80	1.7	2/18 (11.11 %)	M1	S0	E1	T1	C1
4	26	male	16.251	412	7.701	9/19 (47.37 %)	M1	S0	E0	T2	C2
5	39	male	46.533	152	0.149	21/40 (52.5 %)	M1	S0	E0	T0	C0

Note: eGFR (ml/min/1.73m^2) = 175 × (creatinine, mg/dL)$^{-1.234}$ × (age, years) $^{-0.179}$× (if female, ×0.79).

Abbreviation: eGFR, estimated glomerular filtration rate; M, Mesangial hypercellularity; E, Endocapillary hypercellularity; S, Segmental glomerulosclerosis; T, Tubular atrophy/interstitial fibrosis; C, Crescents.
